# Coalescence dynamics in oil-in-water emulsions at elevated temperatures

**DOI:** 10.1038/s41598-021-89919-5

**Published:** 2021-05-26

**Authors:** Bijoy Bera, Rama Khazal, Karin Schroën

**Affiliations:** 1 Food Process and Engineering Group, Wageningen University and Research, Bornse Weilanden 9, 6708 WG Wageningen, The Netherlands; 2 Present Address: Transport Phenomena Group, Department of Chemical Engineering, Delft University of Technology, Van der Maasweg 9, 2629 HZ Delft, The Netherlands

**Keywords:** Condensed-matter physics, Fluid dynamics

## Abstract

Emulsion stability in a flow field is an extremely important issue relevant for many daily-life applications such as separation processes, food manufacturing, oil recovery etc. Microfluidic studies can provide micro-scale insight of the emulsion behavior but have primarily focussed on droplet breakup rather than on droplet coalescence. The crucial impact of certain conditions such as increased pressure or elevated temperature frequently used in industrial processes is completely overlooked in such micro-scale studies. In this work, we investigate droplet coalescence in flowing oil-in-water emulsions subjected to higher than room temperatures namely between 20 to 70 $^{\circ }$C. We use a specifically designed lab-on-a-chip application for this purpose. Coalescence frequency is observed to increase with increasing temperature. We associate with this observation the change in viscosity at higher temperatures triggering a stronger perturbation in the thin aqueous film separating the droplets. Using the scaling law for rupture time of such a thin film, we establish a mechanism leading to a higher coalescence frequency at elevated temperatures.

## Introduction

A huge number of applications in food industry, oil and natural gas industry, separation processes industry require either a stable or unstable oil-in-water emulsion under very specific process conditions^1^. As an example, in an oil–water separation process, temperature is used to facilitate phase separation from the compressed emulsion and hence, coalescence of the oil droplets is desired. On the other hand, high temperature (in short time) treatment of dairy products ensures sterilization of the liquid food and requires non-coalescing dispersed phase at elevated temperatures^2^. In addition, many of these industrial processes require the oil–water mixture to be subjected to flow. The complex interplay of droplet interactions coupled with the process parameters and the flow conditions make such a system extremely difficult to study.

Understanding this system very much requires a description of the micro-scale phenomena since the dispersed phase droplets are of the order of a few hundred nanometers to a few tens of microns. The coverage of the oil–water interface by an emulsifier also plays a significant role in eventual stability of the emulsion and hence, the diffusion of the emulsifier and subsequent adsorption control the behavior and stability of the emulsion at this micro-scale. Prior research has focussed extensively on migration of dispersed phase droplets in a (linear) flow^3^, droplet deformation because of flow^4^, transient effects on the droplet behavior^5^ etc. In the past couple of decades, microfluidics has proven to be an extremely important tool in probing emulsions^6–9^. However, in most of these microfluidic emulsion studies, the focus has remained on droplet breakup (e.g., at a T-junction)^10,11^ i.e., the formation of the emulsion. Both numerical as well as experimental research have been carried out on the dynamics of droplet breakup during the formation of such emulsions^12^. The coalescence studies are, however, mostly limited to determination of droplet size distributions and their analysis^13,14^. This severely limits the understanding of the system since the dynamic flow effects on coalescence cannot be observed using these methods.

Krebs et al. introduced an experimental method for the first time to measure and analyze the coalescence of moving droplets in a microfluidic channel^15^. Surfactant concentration, addition of salt were used as parameters in this study and inferences were drawn regarding the stability of the system. However, such an in-situ microfluidic study of droplet coalescence does not correspond to industrially relevant process conditions. In addition to that, effects of some of the industrial process conditions such as oil volume fraction, droplet size and oil viscosity on the emulsion stability have previously been studied in an empirical fashion^16^, but microscopic insight of what such a specific process condition e.g., elevated temperature does to the thinning of the aqueous film between two droplets leading to the droplets’ coalescence is quite limited.

In our current study, we build a lab-on-a-chip setup where a temperature control is added to the coalescence setup described by Krebs et al. We use two low molecular weight (LMW) surfactants: SDS and Tween-20 (food-grade) as emulsifiers to first form oil-in-water emulsion at the T-junction part of the microfluidic chip. The same emulsion then flows to the part of the microfluidic chip known as coalescence chamber where we study the oil droplets’ coalescence as a function of temperature, surfactant concentration, initial oil droplet size and the adsorption time allowed to the surfactant molecules to get adsorbed to the oil–water interface. Based on the observations, we propose a mechanistic explanation of the film drainage at elevated temperatures and explain how that will lead to the coalescence trends observed in our experiments.

## Methods

### Chemicals

Oil is the dispersed phase in our system and we have used Hexadecane (Sigma Aldrich, The Netherlands) which is filtered using a 30 $\upmu$m microfilter (BD Scientific, The Netherlands). SDS and Tween 20 (Sigma Aldrich) are used as the emulsifiers in these experiments. The continuous phase is prepared by dissolving SDS and Tween 20 in ultrapure water (milli-Q, Merck Millipore, Germany) and subsequently diluting the stock solution to required surfactant concentrations.

**Figure 1:**
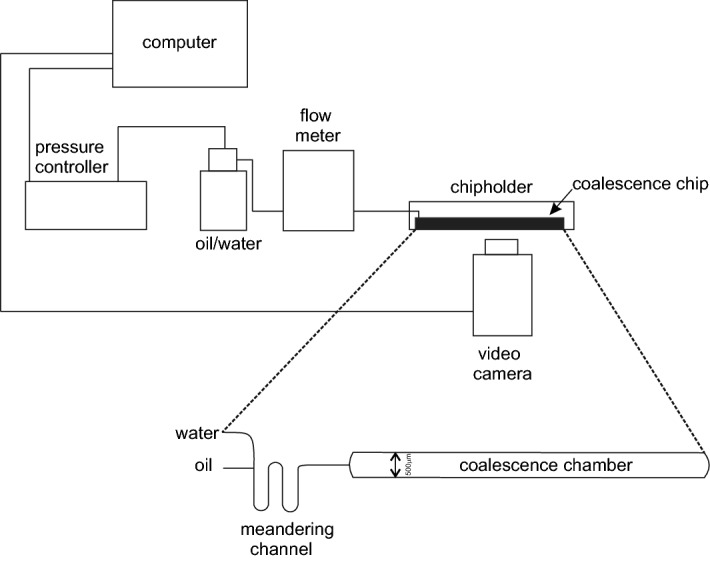
Experimental set-up with the microfluidic coalescence chip shown in inset. The coalescence chip comprises of three parts: the T-junction for formation of droplet and emulsion, the adsorption channel (U-shaped) for surfactant adsorption and finally, the wider coalescence chamber for allowing droplet collision and merging.

### Coalescence setup

The microfluidic chip and setup used in these experiments have been previously used by some of our co-workers^15,17^ and is schematically shown in Fig. 1. In short, a pressure controller (Elveflow, France) is used to inject both hexadecane and surfactant solutions in the chip. The first part of the microfluidic chip consists of a ‘T-junction’, as depicted in Fig. 1, where the oncoming oil phase is sheared by the aqueous surfactant solution. As a result, oil droplets are created inside the microchannel and an oil-in-water emulsion is formed. The emulsion then passes through a winding part in order to allow adsorption of the emulsifier (surfactants) at the oil–water interfaces. In our experiments, the length of this adsorption part of the microchannel is varied between 35 and 100 ms of passage time for the freshly formed emulsion through this part. The following part of the chip is wider (500 $\upmu$m) compared to the rest (100 $\upmu$m). As a result, the oil droplets slow down leading to collision as well as coalescence. This part of the chip is called the coalescence chamber. During the experiments, coalescence occurrences are recorded at the beginning and at the end of this chamber for further analysis. Further details of the chip can be found in^15,17^.

It is important to note that our entire study relies on the dynamic flow conditions achieved in the microfluidic setup. The formation of the oil droplets at the T-junction is possible for a specific ratio of oil phase inflow rate and aqueous phase inflow rate. In addition, any variation in the droplet sizes or the speed with which the droplets arrive at the coalescence chamber, is also a direct result of varying these inflow rates of oil and aqueous phases. Finally, the coalescence events that we measure in our experiments, occur where the continuous phase is flowing around them which would have a significant influence on the drainage of the interstitial films. These flow rates and film drainage are described in greater detail in the later sections.

Before carrying out coalescence experiments, these chips are cleaned thoroughly using a specific cleaning protocol. The chips are first sonicated for 30 min in a 5% DECON solution (Sigma Aldrich, The Netherlands) followed by rinsing and sonication (for 60 min) in milli-Q water. The chip is further sonicated in ethanol before rinsing again with milli-Q water. Subsequently, the chips are baked dry in an oven for 2 h at 500 $^{\circ }$C. After cooling the chip, they are stored in ethanol before use.

### Temperature unit

**Figure 2:**
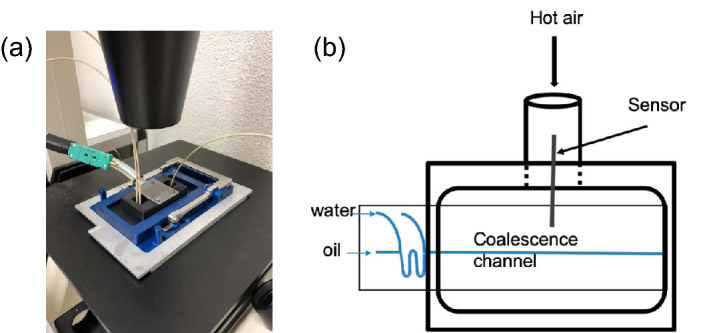
A Lab-ona-chip where temperature can be increased while performing coalescence experiments; (**a**) the actual temperature unit attached to the coalescence chamber at top, (**b**) a schematic explaining the use of heated air to elevate the temperature of the chip.

In order to capture the influence of temperature on the coalescence of oil droplets, a temperature unit is added to the microfluidic setup. As shown in Fig. 2a, a custom made plexiglass holder is placed on top of the coalescence chamber of the chip. The holder is connected to a heating unit with a tube which in turn is connected to the compressed air supply, as shown by the schematic representation in Fig. 2b. Hence, compressed air is let to flow through the heating unit where it can be heated up to 95 $^{\circ }$C. Subsequently, this hot air is blown over the coalescence chamber; a thermostat confirms the temperature of the (outside) of the coalescence chamber, and it it assumed that the inside of the chamber is also at the same temperature.

The usual oil inflow rate at the T-junction is 70 $\upmu$l/min, while the water inflow rate is 200 $\upmu$l/min. We noticed that in order to produce the same size droplets, the flow rates are not required to be changed (significantly) at higher temperatures. We attribute this to the T-junction not being heated directly by the heating element i.e., the change of viscosity at higher temperatures does influence the coalescence of the droplets but not the formation of the droplets which depends on the relative inflow rates. Of course, the whole set-up being in mm-scale, the T-junction is also influenced by the heating element housed on top of the coalescence chamber, but the influence seems not large enough to affect the droplet formation.

### Coalescence experiments inside temperature unit

First, the chip is placed in the temperature controlling holder and then oil and water phases are injected into the chip to form the emulsion. When the oil droplets arrive at the coalescence chamber, a series of images are captured at the beginning of the chamber for a period of 30s and then the same is done at the end of the coalescence chamber as well. The capturing of the coalescence events is started at room temperature (20 $^{\circ }$C) and then subsequently repeated at elevated temperatures up to 70 $^{\circ }$C in steps of 5 $^{\circ }$.

### Calculation of coalescence frequency

The captured images are analyzed following^15,17^ for calculating the frequency of coalescence events. In short, the surface areas of the droplets at the end of the coalescence chamber are calculated from the pixel information from the captured videos. If the mean final droplet volume is given by V$_f$, and the mean initial droplet volume is given by V$_i$, then assuming a monodisperse emulsion before the oil-droplets coalesce (a reasonable assumption for a microfluidic experiment), the mean droplet volume ratio ($\Phi$) is given by:

1 $$\begin{aligned} \Phi =\frac{V_f}{V_i} \end{aligned}$$

Hence, the mean number of coalescence can be calculated by: $N_{coal}=\frac{V_f}{V_i}-1=\Phi -1$. If we assume that a droplet remains for t$_{res}$, the so-called residence time, before reaching the end of the coalescence chamber, then the coalescence frequency is given by:

2 $$\begin{aligned} f_{coal}=\frac{N_{coal}}{t_{res}} \end{aligned}$$

where, t$_{res}$ can be calculated from the mean droplet velocity.

## Results

### Influence of surfactant concentration and temperature

**Figure 3:**
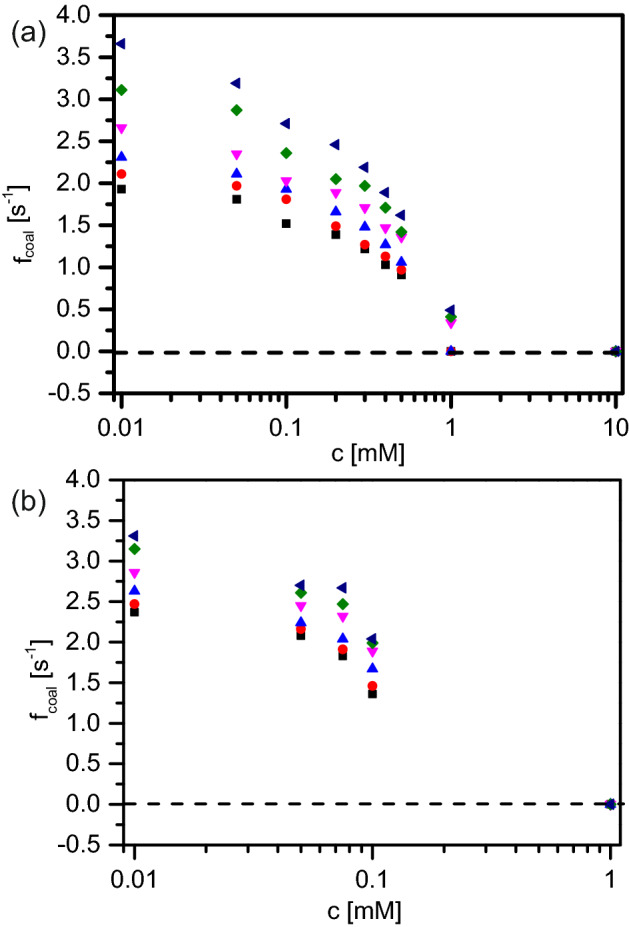
Coalescence trends in hexadecane-in-water with varying concentration of emulsifiers (**a**) SDS, (**b**) Tween 20. Different symbols denote the various temperatures at which the coalescence was measured: 20 $^{\circ }$C (black squares), 30 $^{\circ }$C (red circles), 40 $^{\circ }$C (blue upward triangles), 50 $^{\circ }$C (downward magenta triangles), 60 $^{\circ }$C (green diamonds) and 70 $^{\circ }$C (dark blue leftward triangles). The entire dataset along with the error bars are presented in the supplementary information.

Figure 3a shows the coalescence occurrences with respect to surfactant concentration (0.01–1 mM) when SDS is used as the emulsifier. Temperature is varied between 20 and 70 $^{\circ }$C for each of these SDS concentrations and the figure depicts the entire phase diagram of these coalescence events. We see that coalescence occurrences decrease with increasing SDS concentration (at a constant temperature). As one approaches the critical micellar concentration (cmc) of SDS (8.2 mM), very few oil droplets seem to coalesce. Above the cmc, we do not observe any coalescence. On the other end of the concentration spectrum i.e., at a relatively low SDS concentration ( 0.01 mM), coalescence experiments are difficult to carry out. This is due to most of the oil droplets pinning on the bottom glass of the coalescence chamber. Since the microfluidic chips are cleaned thoroughly before the experiments, the channel is extremely hydrophilic. This, coupled with the fact that hexadecane has a far lower surface tension ($\sim$ 27 mN/m) than that of water ($\sim$ 72 mN/m)^18,19^, implies that unless the surfactant molecules covered the oil-interface efficiently, hexadecane will have a much higher affinity for the (hydrophilic) glass surface. Hence, at lower SDS concentration, hexadecane droplets stick to the bottom glass.

**Figure 4:**
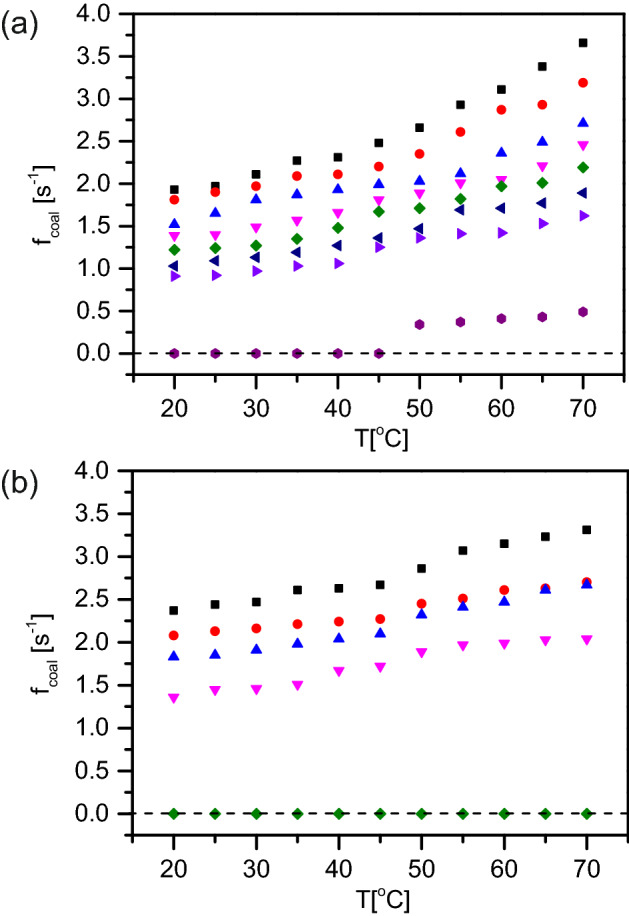
Coalescence trends at elevated temperatures for different (**a**) SDS, (**b**) Tween 20 concentrations. SDS concentrations are: 0.01 mM (black squares), 0.05 mM (red circles), 0.1 mM (blue upward triangles), 0.2 mM (pink downward triangles), 0.3 mM (green diamonds), 0.4 mM (dark blue leftward triangles), 0.5 mM (magenta rightward triangles), 1 mM (purple circles). For Tween 20 the concentrations are: 0.01 mM (black squares), 0.05 mM (red circles), 0.075 mM (blue upward triangles), 0.1 mM (pink downward triangles) and 1 mM (green diamonds). The error bars are shown (along with the entire dataset) in the supplementary information.

Temperature of the coalescence chamber seems to clearly have a significant effect on the coalescence occurrences. With an increasing temperature, we notice an increase in the coalescence events in the microchannel (Fig. 4a). The increase is slower until about 50 $^{\circ }$C and then quite significant up to 70 $^{\circ }$C. This increasing trend is independent of the surfactant concentration. However, below 0.1 mM SDS concentration the coalescence trend with increasing temperature is more erratic. We attribute this to the irregular surface coverage at oil–water interface at low surfactant concentration. It is important to note that all these experiments are carried out at a temperature which is above the Krafft temperature of SDS and hence, the solubility of the surfactant does not directly play a role. This assumption is also validated by the fact that the results presented for each SDS concentration and temperature are average values of a set of three experiments; the errors are reasonably small.

Another surfactant is chosen to examine the generality of these observations, namely Tween 20. Figures 3b and 4b show the coalescence frequency for hexadecane droplets when Tween 20 is used as the emulsifier; the general trend is identical to that of SDS.

### Influence of droplet size

**Figure 5:**
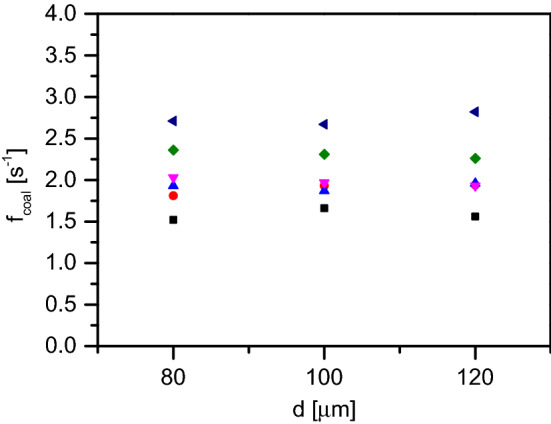
Coalescence trends with different droplet sizes and temperatures at 0.1 mM SDS concentration. Different symbols denote the various temperatures at which the coalescence was measured: 20 $^{\circ }$C (black squares), 30 $^{\circ }$C (red circles), 40 $^{\circ }$C (blue upward triangles), 50 $^{\circ }$C (downward magenta triangles), 60 $^{\circ }$C (green diamonds) and 70 $^{\circ }$C (dark blue leftward triangles).

The size of the dispersed phase droplets is a crucial parameter influencing emulsion stability. In microfluidic experiments, always a monodisperse emulsion is prepared and as a result, each of our coalescence experiments are carried out at a constant oil droplet size (to start with). We have varied the oil droplet sizes between 80 and 120 $\upmu$m using a range of dispersed phase pressure and continuous phase pressure ratios and the coalescence frequency seems not to change significantly based on the oil droplet size. Figure 5 shows a representative measurement with 0.1 mM SDS. It is evident that at room temperature as well as at an elevated temperature, the coalescence frequency remains independent of the droplet size.

**Figure 6:**
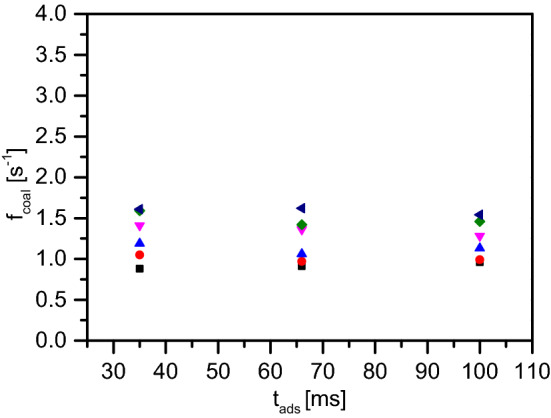
Coalescence trends with different adsorption time: 35 ms, 66 ms and 100 ms, at various temperatures. The data shown here are all at 0.5 mM SDS concentration. Different symbols denote the various temperatures at which the coalescence was measured: 20 $^{\circ }$C (black squares), 30 $^{\circ }$C (red circles), 40 $^{\circ }$C (blue upward triangles), 50 $^{\circ }$C (downward magenta triangles), 60 $^{\circ }$C (green diamonds) and 70 $^{\circ }$C (dark blue leftward triangles).

### Influence of surfactant adsorption time

As explained in the “Materials and methods” section, the microfluidic chip has a meandering section between the T-junction and the coalescence chamber. The length of this meandering section varies from chip to chip, and as a result the time of surfactant adsorption at the oil–water interface can be used as a variable parameter in the experiments, depending on how long the oil drop stays in this section. We have used three different adsorption times (which corresponds to the length of the meandering channels): 35 ms, 66 ms and 100 ms. Figure 6 shows the coalescence frequencies for 0.5 mM SDS concentration (at various temperatures) as the adsorption times are changed. We do not observe a significant change in the coalescence events based on the adsorption time.

## Discussion

For coalescence to occur, two droplets need to approach each other in the microfluidic chip, after which the thin continuous phase film between the droplets needs to drain^20–22^. This drainage of course depends on various flow and fluid properties e.g. drop velocity, fluid viscosity etc. We will first explain the effect of surfactant concentration on this phenomenon.

As the surfactant concentration increases, the oil–water interface has a higher surfactant coverage leading to a lower interfacial tension^23,24^. This implies that less energy is required to form the interface, and as a result it is more advantageous for the oil phase to remain as smaller droplets leading to less coalescence. This is also supported by the fact that the thin film is expected to be more stable when the oil droplet is covered with a surfactant. This is because the film stability is governed by the interaction between the two interfaces (oil–water interface from each droplet) which consists of the van der Waals interactions, the electrostatic interactions and the steric repulsion between the surfactant molecules^25,26^.

**Figure 7:**
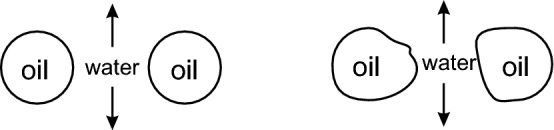
A schematic of two droplets coalescing and the role of capillary wave in the rupture of the aqueous film between these two droplets.

Now, we will focus on the phenomenon of film drainage (leading to droplet coalescence) at elevated temperatures. The fact that such a coalescence event is governed by the capillary velocity is already well-established^20^. In addition, the thin aqueous film squeezed between the two approaching oil droplets is perturbed by the thermal capillary waves (Fig. 7). The resulting fluctuations at the oil–water interface are known to be the most important player in the film rupture^21^. Effectively, the interfacial tensions act to nullify the perturbation at the interface and hence, act as a stabilizing force while the attractive van der Waals forces in the thin aqueous film would like to bring the droplets together and thus act as a destabilizing force. It is the competition of these two counteracting forces that decides the fate of the sandwiched aqueous film.

Capillary time, which is the characteristic time of the decay of the thermal fluctuation at the interface, is given by^27^:

3 $$\begin{aligned} \tau =\frac{\zeta \eta }{\gamma _{ow}} \end{aligned}$$

where, $\zeta$ is the capillary length given by $\zeta =\sqrt{\frac{\gamma _{ow}}{g \Delta \rho }}$ ($\gamma _{ow}$ is the oil–water interfacial tension, *g* is the acceleration due to gravity and $\Delta \rho$ is the density difference between the two phases). $\eta$ is the viscosity of water. Hence, the capillary time can be written as:

4 $$\begin{aligned} \tau =\frac{\eta }{\sqrt{\gamma _{ow} g \Delta \rho }} \end{aligned}$$

From this can be concluded that it is the change in viscosity which impacts the thermal fluctuation time and hence, the rupture time of the thin film more than other parameters. When the temperature is increased from 20 to 70 $^{\circ }$C, the viscosity of water decreases from 1.0025 to 0.423 mPa s, which implies, almost by 60%^28,29^. On the other hand, the tensions at alkane-water interfaces are known to decrease slightly^30^, but nowhere as strongly as the viscosity. As a result, the rupture time of the thin film decreases at elevated temperatures, leading to a higher coalescence frequency.

It is important to note that the change in oil viscosity also indirectly plays a role in this film rupture. The viscosity difference between the two fluids gives rise to a fingering instability at the interface known as the Saffman-Taylor instability^25^. Hexadecane viscosity reduces from 2.56 mPa.s to 1.28 mPa.s when the temperature is increased from 20 to 70 $^{\circ }$C^28^. As a result, it becomes easier for the aqueous phase to form the viscous finger at the oil–water interface and this phenomenon will also impact the drainage of the aqueous phase significantly. The effect of oil viscosity on the coalescence frequency has been previously studied^16^, where a combination of experiments and empirical scaling laws were used to investigate this. The observations in that work can also be supported with our proposed mechanism.

**Figure 8:**
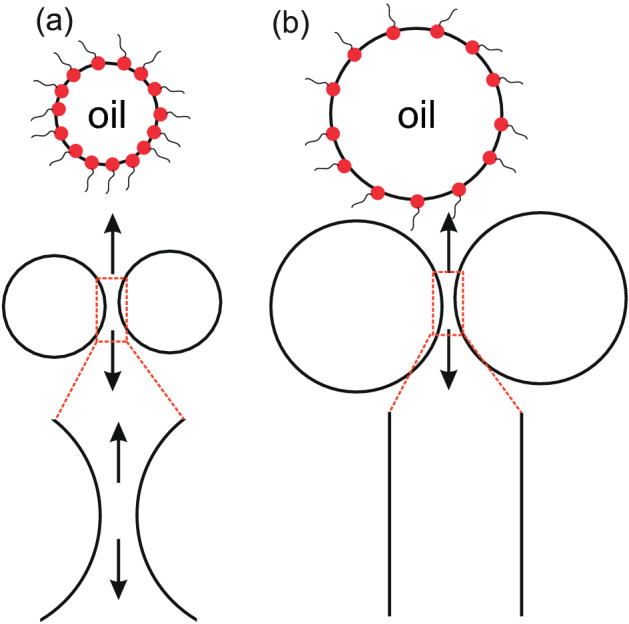
Droplet size seems to have no influence on the coalescence trends (even at elevated temperatures). The plausible explanation is the counteracting effects of droplet coverage (by the surfactant molecules) and the ease of drainage from a flatter film (**a**), compared to that from a more curved film (**b**).

We also notice a significant increase in the coalescence frequency after 50 $^{\circ }$C. This is plausibly due to the formation of micelles which increases around that temperature. The critical micellar concentration (cmc) is known to increase at temperatures around 45 $^{\circ }$C^31^leading to a shift in the equilibrium between monomeric and micellar forms of SDS, hence to an inefficient coverage of the the oil droplets by the SDS molecules and ultimately to easier rupture of the aqueous film between the two oil droplets^32^. In addition, SDS is known to hydrolyze resulting in decomposition and formation of fatty alcohols and sodium sulphate^32^. This would also explain ineffective coverage at oil–water interface by SDS and a subsequent increase in the coalescence.

The following parameter that we changed in our experiment is the droplet size. For three different oil droplet sizes, namely $\sim$ 80 $\upmu$m, $\sim$ 100 $\upmu$m and $\sim$ 120 $\upmu$m, we observed very little or no change in corresponding coalescence frequency at different SDS concentrations and at elevated temperatures. We propose two conteracting influences leading to this result. As the droplet size increases, the oil–water interface is more scarcely covered by the surfactant molecules (at the same surfactant concentration), as shown in Fig. 8a; this should lead to a higher coalescence frequency. On the other hand, bigger droplets would lead to a flatter aqueous film sandwiched between two such droplets, as shown in Fig. 8b; such a flat film is more stable^20^. Bigger droplets of course have a higher expansion pressure, but seeing that we allowed equal amount of time at the T-junction to form droplets irrespective of their sizes, we believe that these two counteracting phenomena balance each other so that the coalescence frequency remains constant. It is important to note that during these experiments, the droplet number density is kept constant (10,000 drops passing the region of interest in 33 s) even when the droplet sizes is changed. As a result, an increase in droplet size leads to an increase in oil volume fraction, which in turn increases the interfacial area per unit volume of the emulsion. The droplet size is increased while keeping the droplet number density constant by changing the oil and continuous phase injection pressures proportionally.

One needs to pay attention to the type of equilibrium that we consider in this explanation. If we consider the initial surface coverage of the drop, then the bigger droplet will have a lower surface coverage since it has a faster expanding surface. At a later stage, the equilibrium between the oil–water interface and the bulk (aqueous) solution is strongly related to the bulk concentration. At a relatively high surfactant concentration (around the c.m.c.), one can expect that same number of surfactant molecules will go to a smaller or a larger droplet. However, the surfactant concentrations at which we observe coalescence are far below the cmc of SDS. As a result, the equilibrium between the interface and the bulk will act as a driving factor for an increase in the surface coverage.

It is noteworthy, that in food industry, separation processes industry and petrochemical industry, the range of oil droplet sizes is very wide. Starting from a few nms large oil droplets (in food grade emulsions), the size of dispersed phase droplets can go to as high as a few hundred $\upmu$ms. In order to vary the droplet size significantly in an experiment such as ours, one need to introduce new design of the T-junctions in the microfluidic channels where the ratio of oil and water flow rates create the dispersed phase droplets. To keep the focus of our work intact (i.e., specific process conditions affecting the droplet dynamics), we have limited our use of microfluidic chip to a single T-junction design.

Finally, the fact that the coalescence frequency seems to be independent of the adsorption time allowed to the surfactant molecules depends possibly on the short adsorption time of such molecules. The time required by such a low molecular weight (LMW) surfactant molecule to adsorb at the oil–water interface can be represented by the Ward–Tordai equation^24^:

5 $$\begin{aligned} t_{ads}=\frac{\Gamma ^2}{c_{b}^2 \cdot D} \end{aligned}$$

where, $\Gamma$ is the surface excess coverage, $c_b$ is the bulk concentration and *D*, the diffusion constant. Assuming a simple Langmuir isotherm^15^, we can write:

6 $$\begin{aligned} \Gamma&=\frac{\Gamma _\infty \cdot c_b}{(c+c_b)} \nonumber \\ \implies t_{ads}&=\frac{\Gamma _\infty ^2 \cdot c_b^2}{(c+c_b)^2 c_b^2 \cdot D} \nonumber \\ \implies t_{ads}&=\frac{\Gamma _\infty ^2}{(c+c_b)^2 \cdot D} \end{aligned}$$

Inserting typical values of maximum surface coverage $\Gamma _\infty =3.75 \times 10^{-6}$ mol/m$^{-2}$, local concentration $c=1.73 \times 10^{-2}$ mol/m$^3$ and diffusion constant $D=10^{-10}$ mol/m$^2$ from Ref.^24^, we see that even at 1 mM bulk SDS concentration, $t_{ads}$ is much lower than the minimum adsorption time (35 ms) in our microfluidic chip. This is why we do not observe a direct effect of the meandering channel on the coalescence frequency.

## Conclusion

We have carried out unique experiments where emulsion stability is investigated at elevated temperature, to the best of our knowledge, first time in micro-scale. Using a custom-made microfluidic setup which is heated to a specific temperature, oil-in-water emulsion is formed at a T-junction microchannel and in the same chip, the coalescence between the oil droplets is studied. When a low molecular weight surfactant is used as the emulsifier, the oil droplets coalesce more frequently as the temperature is increased and these droplets coalesce less frequently when the surfactant concentration is increased. Due to micellization the increase in coalescence is more pronounced after 50 $^{\circ }$C. Thermal fluctuations at the interface and the contribution of viscosity are identified as the key players in expediting the drainage and subsequent rupture of the thin aqueous film between to coalescing droplets. The droplet size and the surfactant adsorption time do not play a big role in such a monodisperse emulsion, at least within the process conditions that we have probed in this work. In our opinion, these results show that the microfluidic tools used in our investigation are versatile and able to quantify differences in coalescence stability in a systematic way. We consider this the first step towards understanding the effects to processes as carried out on large scale in industry.

## Supplementary Information

Supplementary Information.
