# Supplementary material for: Coalescence dynamics in oil-in-water emulsions at elevated temperatures
Source: Sci Rep. 2021 May 26;11:10990. doi: 10.1038/s41598-021-89919-5 (PMC8155042; doi:10.1038/s41598-021-89919-5)
Supplement: Supplementary file 1 — Supplementary Information. [file 41598_2021_89919_MOESM1_ESM.pdf]

Supplementary Information: Coalescence dynamics in oil-in-water emulsions at elevated temperatures

Bijoy Bera, Rama Khazal, Karin Schroën

December 23, 2020

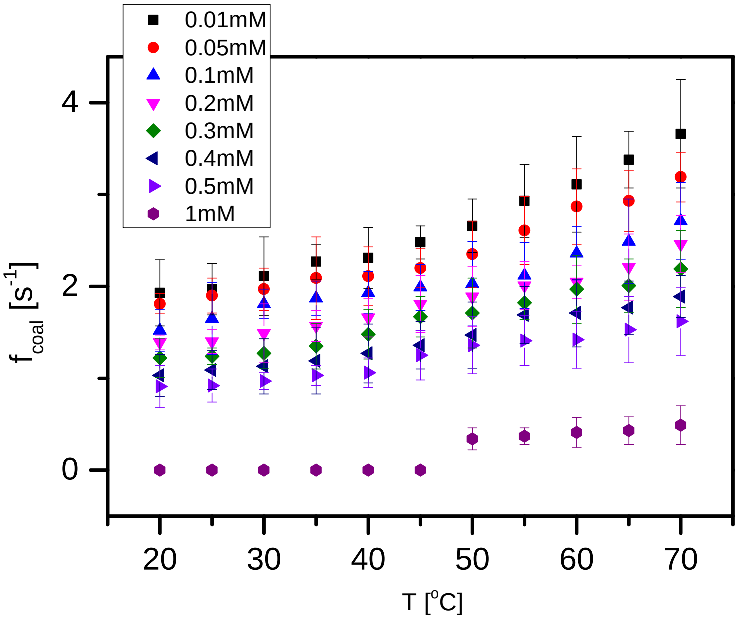

Figure 1: Complete coalescence picture of SDS.

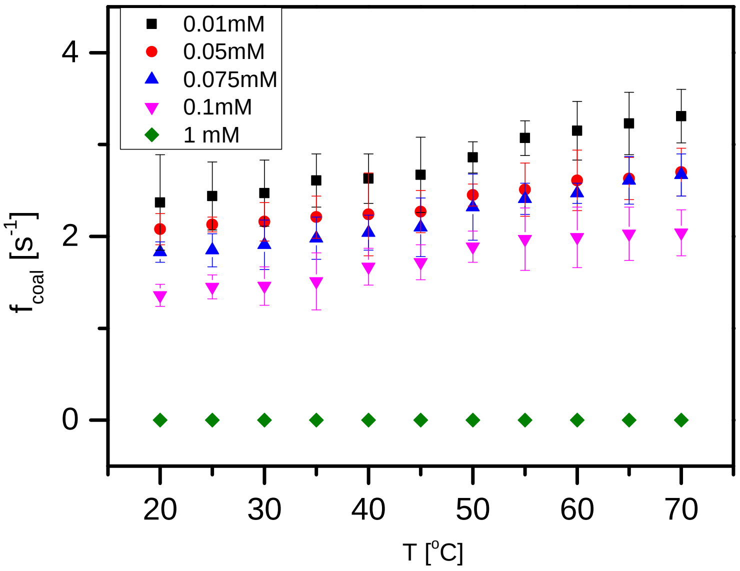

Figure 2: Complete coalescence picture of Tween 20.
